# Two-Component Signaling Regulates Osmotic Stress Adaptation via SskA and the High-Osmolarity Glycerol MAPK Pathway in the Human Pathogen *Talaromyces marneffei*

**DOI:** 10.1128/mSphere.00086-15

**Published:** 2016-02-24

**Authors:** Kylie J. Boyce, Cunwei Cao, Alex Andrianopoulos

**Affiliations:** a School of Biosciences, The University of Melbourne, Parkville, Victoria, Australia; b Department of Dermatology and Venereology, First Affiliated Hospital of Guangxi Medical University, Nanning, China; Karlsruhe Institute of Technology (KIT)

**Keywords:** Two-component signaling, dimorphism, morphogenesis, pathogenicity, response regulator

## Abstract

For successful infection to occur, a pathogen must be able to evade or tolerate the host’s defense systems. This requires the pathogen to first recognize the host environment and then signal this response to elicit a complex adaptive program in order to activate its own defense strategies. In both prokaryotes and eukaryotes, two-component signaling systems are utilized to sense and respond to changes in the external environment. The hybrid histidine kinases (HHKs) at the start of the two-component signaling pathway have been well characterized in human pathogens. However, how these HHKs regulate processes downstream currently remains unclear. This study describes the role of a response regulator downstream of these HHKs, *sskA*, in *Talaromyces marneffei*, a dimorphic human pathogen. *sskA* is required for asexual reproduction, hyphal morphogenesis, cell wall integrity, osmotic adaptation, and the morphogenesis of yeast cells both *in vitro* at 37°C and during macrophage infection, but not during dimorphic switching. Comparison of the Δ*sskA* mutant with a strain in which the mitogen-activated protein kinase (MAPK) of the high-osmolarity glycerol pathway (SakA) has been deleted suggests that SskA acts upstream of this pathway in *T. marneffei* to regulate these morphogenetic processes. This was confirmed by assessing the amount of phosphorylated SakA in the Δ*sskA* mutant, antifungal resistance due to a lack of SakA activation, and the ability of a constitutively active *sakA* allele (*sakA^F316L^*) to suppress the Δ*sskA* mutant phenotypes. We conclude that SskA regulates morphogenesis and osmotic stress adaptation in *T. marneffei* via phosphorylation of the SakA MAPK of the high-osmolarity glycerol pathway.

**IMPORTANCE** This is the first study in a dimorphic fungal pathogen to investigate the role of a response regulator downstream of two-component signaling systems and its connection to the high-osmolarity glycerol pathway. This study will inspire further research into the downstream components of two-component signaling systems and their role during pathogenic growth.

## Abstract (precis)

This is the first study in a dimorphic fungal pathogen to investigate the role of a response regulator downstream of two-component signaling systems and its connection to the high-osmolarity glycerol pathway. This study will inspire further research into the downstream components of two-component signaling systems and their role during pathogenic growth.

## INTRODUCTION

The abilities of pathogenic bacteria, parasites, and fungi to invade and survive within mammalian hosts are governed by a complex interaction between the microbe and the host. It is essential for pathogens to evade or tolerate the host’s defense systems in order to allow successful infection to occur. The pathogen is required to recognize the host environment and subsequently signal to elicit changes in gene expression in order to activate its own defense strategies. Two-component signaling systems are utilized in both prokaryotes and eukaryotes to sense and respond to changes in the external environment and are essential for virulence (reviewed in references 1, 2, and 3). In bacteria, two-component signaling systems consist of a membrane-associated sensor histidine kinase (HK) and a cytoplasmic response regulator (RR) protein. An environmental signal triggers the phosphorylation of a conserved histidine residue in the HK kinase domain, and this phosphorylation signal is relayed to a conserved aspartic acid residue in the effector domain of the RR, which mediates changes in gene expression. In fungi, the HK and RR are fused (hybrid HK [HHK]), and additional phosphorelay occurs via a phosphotransmitter protein (HPt) and a second RR. The RR either directly regulates gene expression or activates a mitogen-activated protein kinase (MAPK) pathway that in turn regulates gene expression.

*Saccharomyces cerevisiae* possesses a single HHK (class VI) and HPt, encoded by *SLN1* and *YPD1*, respectively, and two response regulators, encoded by *SSK1* and *SKN7* (reviewed in reference 4). In response to oxidative stress, Skn7p binds directly to DNA to regulate changes in gene expression (5). Skn7p binds to DNA regardless of its phosphorylation state; however, expression of *SLN1*-dependent genes occurs only in response to receiver domain phosphorylation by Sln1p (6, 7). In the absence of stress, Ssk1p negatively regulates the high-osmolarity glycerol (HOG) MAPK signaling pathway. Under hyperosmotic conditions, the kinase domain of Sln1p is inactive, resulting in a cessation of the Sln1p-Ypd1p-Ssk1p phosphorelay. This leads to the rapid dephosphorylation of Ssk1p, which then binds the redundant Ssk2p and Ssk22p MAPKKKs (MAPK kinase kinases), leading to activation of the MAPKK Pbs2p, which activates the MAPK Hog1p (reviewed in reference 8). Pbs2p can also be activated independently of Sln1p by the Sho1p osmosensor via phosphorylation of the Ste11p MAPKKK (9). Ste11p activation is thought to occur by the Ste20p p21-activated protein kinase (PAK) and also involves Cdc42p and Ste50p (reviewed in reference 8). Phosphorylated Hog1p interacts with a number of transcription factors, including Hot1p, Msn2/4p, Sko1p, and Smp1p responsible for the induction of genes required for the response to osmotic stress (10–12). In addition to direct phosphorylation of transcription factors, Hog1p also controls gene expression by chromatin association and by recruiting RNA polymerase II (RNA Pol II) machinery and histone deacetylases to promoters (reviewed in reference 8).

Fungi with more-complex life cycles have a greatly expanded set of HHKs that transmit the phosphorylation signal via a single HPt, orthologous to Ypd1p, and at least two RRs, orthologous to Ssk1p and Skn7p (13). HHKs have been comprehensively investigated in plant pathogens due to mutations in the class III HHKs resulting in reduced pathogenicity and increased resistance to dicarboximide and phenylpyrrole antifungal agents, which act by Hog1-induced glycerol accumulation in the absence of high external osmolarity (14–21). In human pathogens, class III HHKs play conserved roles in fungicide resistance, pathogenicity, and osmotic and oxidative stress adaptation, in addition to regulating other processes required for virulence in a mammalian host such as melanin production, cell wall integrity, mating, dimorphism, and the production of infectious propagules through asexual development (22–24). However, how pathway components downstream of the HHKs act to regulate these processes in human-pathogenic fungi, especially those capable of dimorphic growth, currently remains unclear.

*Talaromyces marneffei* (formerly called *Penicillium marneffei*) is a dimorphic human pathogen prevalent in South-East Asia. Like many other human pathogens, *T. marneffei* exhibits thermally regulated dimorphic switching such that it grows as highly polarized multicellular hyphae at 25°C and switches to a unicellular yeast growth form at 37°C. Deletion of the HHKs encoded by *drkA* (class III) and *slnA* (class VI, *SLN1* orthologue) has revealed that these HKs have both overlapping and unique roles in *T. marneffei*, with DrkA playing a more significant role during growth and development. DrkA, and to a lesser extent SlnA, plays a role in asexual development, hyphal morphogenesis, and cell wall integrity (23). SlnA has a unique role during conidial germination, whereas DrkA is essential for yeast growth at 37°C, both *in vitro* and *ex vivo*, antifungal resistance, and phosphorylation of the MAPK SakA (orthologous to *S. cerevisiae* Hog1p) during osmotic stress adaptation (23). This study describes the role of a response regulator downstream of these HHKs, *sskA* (homologous to *S. cerevisiae* Ssk1p) in *T. marneffei*. Like DrkA, *sskA* is required for the production and viability of asexual spores, hyphal morphogenesis, cell wall integrity, osmotic adaptation, and the morphogenesis of yeast cells both *in vitro* at 37°C and during macrophage infection, but not during dimorphic switching. Studies using *sskA* and *sakA* mutants show that there is a lack of phosphorylated SakA in the Δ*sskA* mutant. In addition, the Δ*sskA* mutant is resistant to the dicarboximide and phenylpyrrole classes of antifungals, and a constitutively active *sakA* allele (*sakA^F316L^* [the F-to-L change at position 316 encoded by *sakA*]) can suppress the Δ*sskA* mutant phenotypes. These data provide strong evidence that SskA acts upstream of SakA to regulate these processes in *T. marneffei*.

## RESULTS

### The *T. marneffei* genome encodes four putative response regulators.

**FIG 1:**
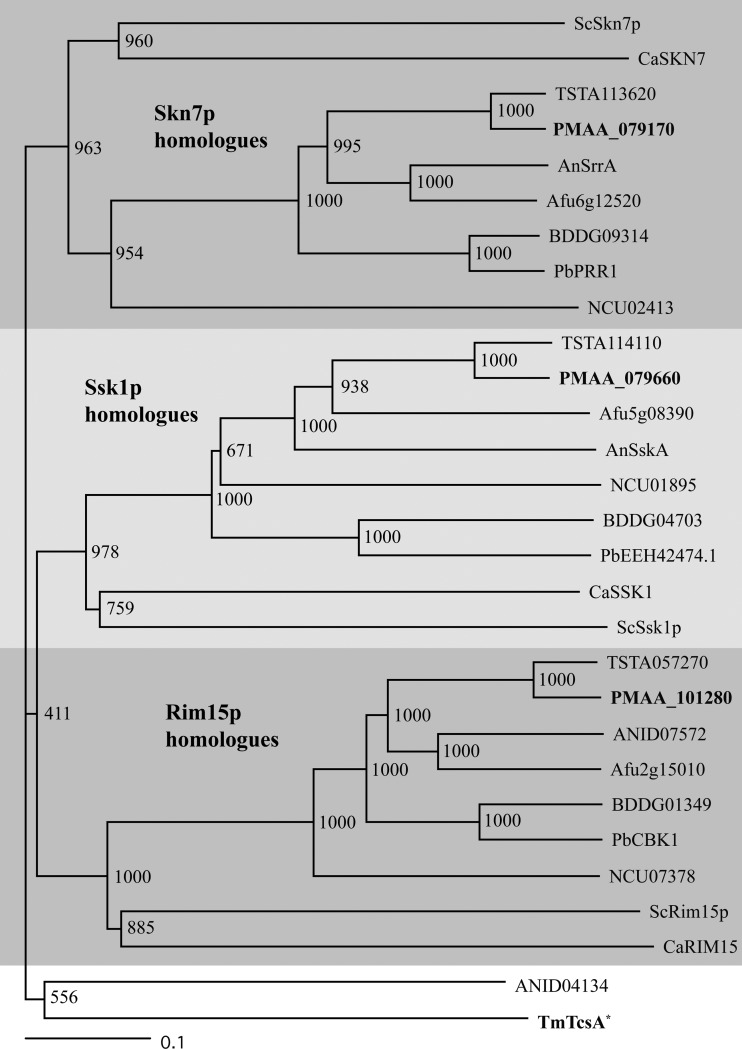
The *T. marneffei* genome encodes four putative response regulators. The bootstrapped relatedness tree was generated using ClustalX with protein sequences from *Saccharomyces cerevisiae* (Sc), *Candida albicans* (Ca), *Talaromyces stipitatus* (TSTA), *Talaromyces marneffei* (Tm), *Aspergillus nidulans* (An), *Aspergillus fumigatus* (Afu), *Blastomyces dermatitidis* (BDD), *Paracoccidioides brasiliensis* (Pb), and *Neurospora crassa* (NCU). Skn7p, Ssk1p, and Rim15p homologues are indicated by gray shading. *T. marneffei* SrrA, SskA, RimA, and TcsA* are highlighted in bold. The asterisk after TmTcsA* indicates that the protein is truncated. The scale bar at bottom indicates the length of branch that represents an amount genetic change of 0.1.

The *T. marneffei* genome encodes four putative response regulators (RRs) containing response regulator receiver domains (PFam00072) (GenBank accession no. NW_002196663.1, PMAA_079660; NW_002196663.1, PMAA_079170; NW_002196664.1, PMAA_101280; and NW_002196665.1, PMAA_053050). PMAA_079660 and PMAA_079170 show strong sequence homology to *S. cerevisiae* Ssk1p and Skn7p, respectively (Fig. 1). PMAA_101280 shows sequence homology with *S. cerevisiae* Rim15p (Fig. 1). Rim15p is a kinase required for cellular proliferation in response to nutrients (25). Rim15p homologues lack the conserved aspartic acid residue in the effector domain found in canonical RRs, such as Ssk1p and Skn7p. The presence of a response regulator receiver domain at residues 220 to 330 in PMAA_053050 initially indicated that this gene encoded a unique response regulator in *T. marneffei*. However, comparison of the genomic location encompassing PMAA_053050 with that of a close sexual relative *Talaromyces stipitatus*, revealed the presence of the *tcsA* HHK homologue (TSTA_019980) in the equivalent genomic location in *T. stipitatus*. The genomic synteny extended on both sides of this location, and the *T. marneffei* genome lacked a *tcsA* homologue elsewhere. Subsequent alignment of PMAA_053050 with homologues of *tscA* from *T. stipitatus*, *Neurospora crassa*, *Blastomyces dermatitidis*, and *Aspergillus nidulans* confirmed that PMAA_053050 encodes a truncated form of *tcsA* lacking the HATPase_c (PF02518) and HisKA (PF00512) histidine kinase domains*.* Therefore, *T. marneffei* possesses two canonical RRs homologous to Ssk1p and Skn7p and one noncanonical RR homologous to Rim15p.

### *sskA* is required for the production and viability of asexual spores.

To investigate the role of *sskA* in *T. marneffei*, PMAA_032020 was cloned, and a deletion strain (Δ*sskA*::*pyrG^+^* [strain G991]) was generated by transforming *T. marneffei* strain G816 (Δ*ligD niaD1 pyrG1*) (26). To generate a complemented strain, the Δ*sskA* strain was transformed with an *sskA^+^* fragment targeted to the *niaD* locus. However, no transformants could be isolated by using this protoplast method, most likely due to osmosensitivity in this strain. Control transformation plates (minus selection) showed a 10-fold reduction in Δ*sskA* protoplast regeneration. Therefore, a strain was generated in which *sskA^+^* was targeted to *niaD* (strain G1018). This strain was transformed with the Δ*sskA* deletion construct to generate Δ*sskA sskA^+^* (strain G1019). This strain exhibited a wild-type phenotype (see Fig. S1A in the supplemental material). To confirm that *sskA^+^* targeting *niaD* was complementing the Δ*sskA* phenotype, the Δ*sskA sskA^+^* strain was plated on chlorate to select for the loss of the *sskA^+^* plasmid integrated at *niaD*. Removal of *niaA^t^ sskA^+^* resulted in a strain displaying the Δ*sskA* phenotype (Fig. S1B).

10.1128/mSphere.00086-15.1 Figure S1  Complementation of Δ*sskA*, Δ*mpkA*, and Δ*mpkB* strains. (A) Colonies of a wild-type control strain (Δ*ligD pyrG^t^*), Δ*sskA sskA^+^*, Δ*mpkA mpkA^+^*, and Δ*mpkB mpkB^+^* strains grown on ANM plus supplements plus GABA at 25°C for 14 days. (B) The Δ*sskA sskA^+^* strain was plated on chlorate to select for the loss of the *sskA^+^* plasmid integrated at *niaD* and then grown for 14 days at 25°C on 1% ANM plus GABA plus various stress-inducing agents (calcofluor white [CAL], sodium dodecyl sulfate [SDS], Congo red [CR], sorbitol, and sodium chloride [NaCl]). Removal of *niaA^t^ sskA^+^* resulted in a strain displaying the Δ*sskA* phenotype. (C) The wild-type, Δ*sskA sskA^+^*, Δ*mpkA mpkA^+^*, and Δ*mpkB mpkB^+^* strains were grown for 14 days at 25°C on 1% ANM plus GABA plus various stress-inducing agents (calcofluor white [CAL], sodium dodecyl sulfate [SDS], Congo red [CR], hydrogen peroxide [H_2_O_2_], sorbitol, potassium chloride [KCl], and sodium chloride [NaCl]). Download Figure S1, TIF file, 7 MB. Copyright © 2016 Boyce et al. 2016 Boyce et al. This content is distributed under the terms of the Creative Commons Attribution 4.0 International license.

To assess what functions of *T. marneffei* SskA are independent of MAPK signaling pathways, the phenotype of the Δ*sskA* mutant was compared to the phenotypes of strains in which the genes encoding one of the three MAPKs was deleted. Deletions of the three MAPKs in *T. marneffei*, encoded by *sakA* (GenBank accession no. NW_002196662.1, PMAA_067980), *mpkA* (NW_002196663.1, PMAA_080160), and *mpkB* (unannotated in genome sequence) were generated by transforming *T. marneffei* strain G816 (Δ*ligD niaD1 pyrG1*) (26). Complemented strains (Δ*mpkA mpkA^+^* [strain G959] and Δ*mpkB mpkB*^+^ [strain G1022]) were generated by transforming Δ*mpkB pyrG^−^* (G958) and Δ*mpkB pyrG^−^* (G961) strains with either an *mpkA* or *mpkB* fragment targeted to the *pyrG* locus*.*

**FIG 2:**
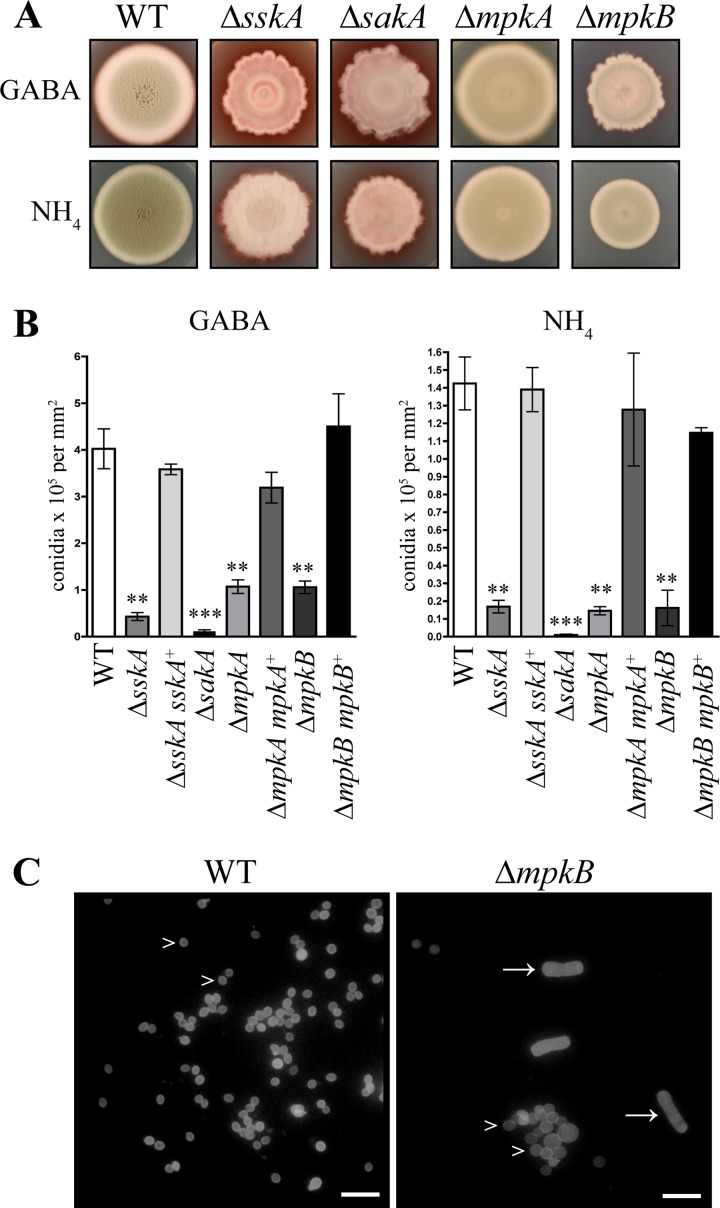
Deletion of *sskA* and genes encoding MAPKs results in reduced asexual development at 25°C. (A) Colonies of a wild-type (WT) control strain (Δ*ligD pyrG^t^*) and Δ*sskA*, Δ*sakA*, Δ*mpkA*, and Δ*mpkB* strains grown on ANM plus supplements plus GABA or ammonium (NH_4_) at 25°C for 14 days. (B) Quantification of the conidial density (conidia × 10^5^ per mm^2^) of strains grown on either GABA or ammonium as the sole nitrogen source after 10 days at 25°C. Statistical significance using an unpaired *t* test and 95% confidence interval is indicated by asterisks as follows: **, *P* < 0.005; ***, *P* < 0.001. (C) Conidial preparations of the wild type and the Δ*mpkB* mutant grown on ammonium as a nitrogen source after 10 days at 25°C. The Δ*mpkB* mutant inappropriately produces yeast cells (white arrows) in addition to conidia (white arrowheads). Images with epifluorescence to observe calcofluor white-stained fungal cell walls are shown. Bars, 10 µm.

At 25°C, *T. marneffei* grows as highly polarized vegetative hyphae that form a branched network called a mycelium. Hyphae undergo asexual development, a process that involves cellular differentiation into asexual structures (conidiophores) and culminates in the production of pigmented asexual spores (conidia). After 14 days at 25°C, the Δ*sskA* strains showed a drastic reduction in the production of asexual development structures. In contrast to the green colonies of the wild type, the Δ*sskA* colonies appeared pink due to a lack of mature pigmented conidia (Fig. 2A). The Δ*sskA* strains produced only a small number of conidia in an irregular pattern in the center of the colony and as a ring around the colony center (Fig. 2A). In addition, unlike the circular colonies of the wild type, the Δ*sskA* mutant colonies exhibited an irregular shape with nonuniform edges (Fig. 2A). The Δ*sskA* mutant colonies also excreted red pigment into the medium. This phenotype was complemented in the Δ*sskA sskA*^+^ strain (see Fig. S1A in the supplemental material). To quantify the decrease in conidiation observed in the Δ*sskA* mutant, 1 × 10^5^ conidia were spread on an agar plate with either ammonium or gamma-aminobutyric acid (GABA) as the nitrogen source, and the conidial density was calculated after 10 days at 25°C. The Δ*sskA* mutant showed a statistically significant decrease in conidial density compared to the wild type and the Δ*sskA sskA^+^* strain (Fig. 2B). The Δ*sakA* mutant colonies showed a phenotype indistinguishable from that of the Δ*sskA* mutant (Fig. 2A). Similar to the Δ*sskA* mutant, the Δ*sakA* mutant also showed a statistically significant decrease in conidial density, but in this case it was even more pronounced (Fig. 2B). Higher osmolarity can often remediate defects in conidiation rate in a number of fungi; however, growth of the Δ*sskA* and Δ*sakA* mutant*s* on 0.5 M sorbitol at 25°C for 14 days did not restore visible conidiation (see Fig. 5). Both the Δ*mpkA* and Δ*mpkB* mutants also showed a statistically significant decrease in conidiation compared to the wild type and the respective complemented strains, although not to the same extent as the Δ*sskA* and Δ*sakA* mutants (Fig. 2A and B). On GABA but not ammonium, the Δ*mpkB* strain also produced conidia in an irregular pattern in the center of the colony and as a ring around the colony center and showed an irregularly shaped colony with nonuniform edges (Fig. 2A). It was also apparent during the isolation of conidia for this analysis that the Δ*mpkB* mutant inappropriately produced yeast cells in addition to conidia at 25°C (Fig. 2C). This phenotype was observed only when the Δ*mpkB* mutant was grown on ammonium as the nitrogen source, not GABA, and did not affect the decrease in conidial density (Fig. 2B). Colonies of the Δ*mpkA mpkA^+^* and Δ*mpkB mpkB^+^* strains were indistinguishable from the wild-type colonies (Fig. S1A).

**FIG 3:**
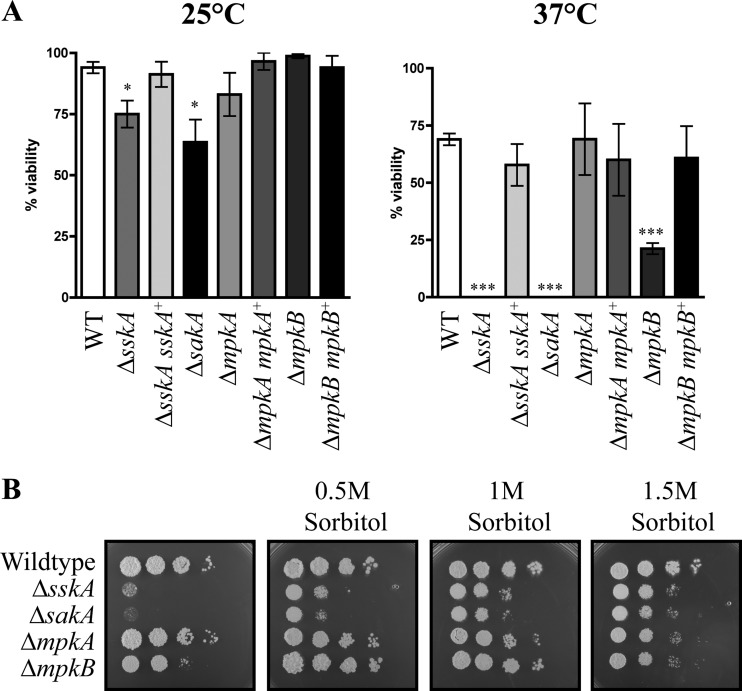
*sskA* and *sakA* are essential for conidial viability at 37°C. (A) Quantification of the percentage of viable conidia at 25°C and 37°C. Statistical significance using an unpaired *t* test and 95% confidence interval is indicated by asterisks as follows: *, *P* < 0.05; ***, *P* < 0.0001. (B) Conidial suspensions of the wild-type, Δ*sskA*, Δ*sakA*, Δ*mpkA*, and Δ*mpkB* strains were dropped onto plates containing synthetic dextrose (SD) plus (NH_4_)_2_SO_4_ and 0.5 M, 1 M, or 1.5 M sorbitol and incubated for 5 days at 37°C.

In addition, the viability of conidia was tested at both 25°C and 37°C (Fig. 3A). In contrast to the wild-type, Δ*mpkA*, and complemented strains, the Δ*sskA* and Δ*sakA* conidia showed a small reduction in conidial viability at 25°C and a severe reduction in viability at 37°C (Fig. 3A). These reductions were statistically significant. A statistically significant decrease in conidial viability was also observed in the Δ*mpkB* strain at 37°C but not 25°C (Fig. 3A). The decreases in viability at 37°C could be remediated by the addition of increasing concentrations of sorbitol (Fig. 3B).

To investigate whether conidiophores possessed wild-type morphology, the wild-type, Δ*sskA*, Δ*sskA sskA^+^*, Δ*sakA*, Δ*mpkA*, Δ*mpkA mpkA^+^*, Δ*mpkB*, and Δ*mpkB mpkB^+^* strains were grown on 4 days at 25°C and stained with calcofluor white to visualize cell walls. Wild-type *T. marneffei* produces conidiophores from a specialized stalk from which differentiated cells are produced sequentially in a budding fashion: metulae bud from the stalk, phialides bud from metulae, and uninucleate conidia bud from phialides. The conidiophores of all strains displayed wild-type morphology (data not shown).

### The Δ*sskA* mutant displays defects in hyphal morphogenesis and cell wall integrity.

**FIG 4:**
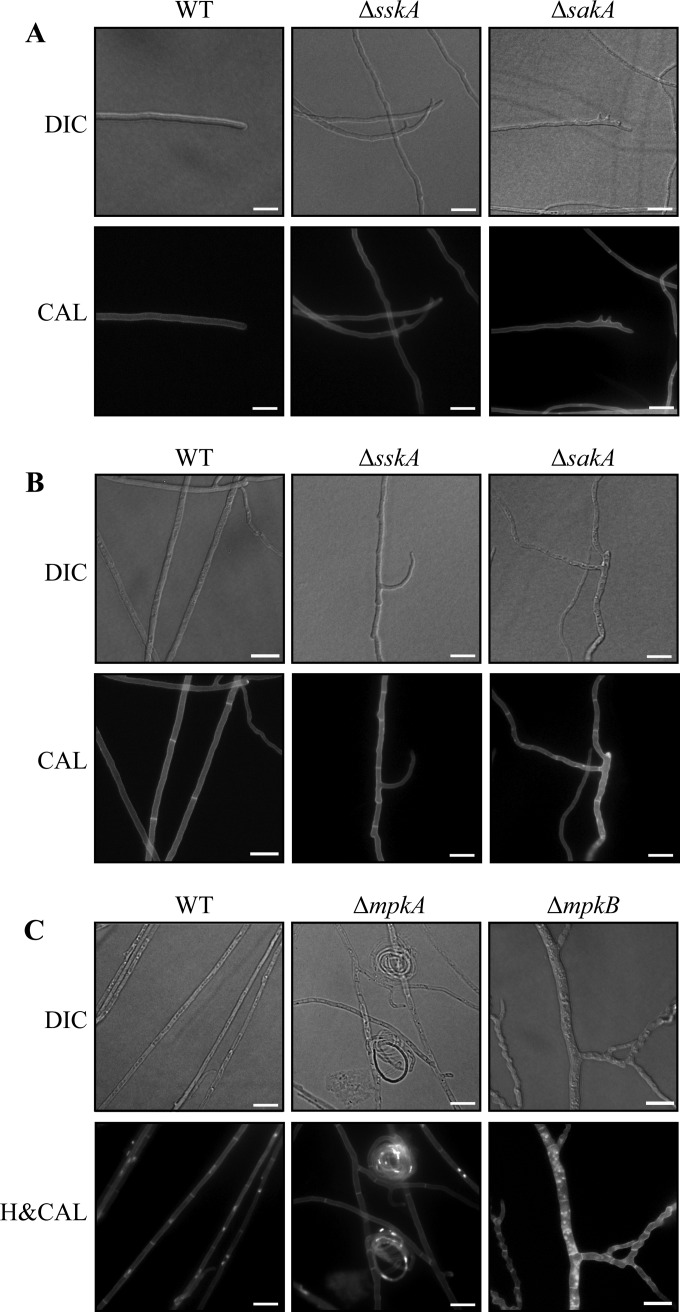
Deletion of *sskA* and genes encoding MAPKs results in defects in hyphal morphology at 25°C. (A to C) The wild-type, Δ*sskA*, Δ*sakA*, Δ*mpkA*, and Δ*mpkB* strains were grown for 4 days at 25°C on 1% ANM plus GABA (A and B) or (NH_4_)_2_SO_4_ (C) and stained with calcofluor white (CAL) to visualize cell walls and septa (A to C) and Hoechst 33258 to visualize nuclei (C). (A) Apical cells of the Δ*sskA* and Δ*sakA* mutants can display dichotomous branching. (B) Subapical hyphal cells of the Δ*sskA* and Δ*sakA* mutants display aberrant branching, cellular swellings, and abnormal chitin deposits. (C) Subapical hyphal cells of the Δ*mpkA* and Δ*mpkB* mutants. The Δ*mpkA* mutant exhibited cell lysis and produced tightly coiled balls of hyphae. Δ*mpkB* hyphae were swollen and bumpy, exhibited dichotomous branching, and were extremely multinucleate. Images were captured using differential interference contrast (DIC) or with epifluorescence to observe fungal cell walls stained with calcofluor white (CAL) or nuclei stained with Hoechst 33258 (H&CAL). Bars, 10 µm.

The Δ*sskA*, Δ*sakA*, and Δ*mpkB* mutants exhibited irregularly shaped colonies with nonuniform edges (Fig. 2A). To investigate whether these colonial phenotypes are a result of defects in hyphal morphogenesis, the wild-type, Δ*sskA*, Δ*sskA sskA^+^*, Δ*sakA*, Δ*mpkA*, Δ*mpkA mpkA^+^*, Δ*mpkB*, and Δ*mpkB mpkB^+^* strains were grown for 4 days at 25°C and stained with either calcofluor white to visualize cell walls or Hoechst 33258 to observe nuclei. Wild-type *T. marneffei* grows as septate, branched hyphae (Fig. 4) of which subapical cells are predominately uninucleate, whereas apical cells are multinucleate (Fig. 4C). The Δ*sskA* and Δ*sakA* mutants displayed subtle defects in hyphal morphology such as hyphal curling and dichotomous branching in some apical cells (Fig. 4A) as well as abnormal bumps and chitin deposits along hyphae (Fig. 4B) compared to all the other strains. The Δ*mpkA* mutant produced numerous tightly coiled balls of hyphae, and cell lysis was also evident (Fig. 4C). Interestingly, Δ*mpkB* hyphae appeared wild type when grown on GABA as the nitrogen source, but hyphae displayed aberrant morphology when grown on ammonium as the nitrogen source (Fig. 4C). Hyphae were swollen and bumpy, exhibited dichotomous branching, and were extremely multinucleate (Fig. 4C). The Δ*sskA sskA^+^*, Δ*mpkA mpkA^+^*, and Δ*mpkB mpkB^+^* strains were indistinguishable from the wild type (data not shown).

**FIG 5:**
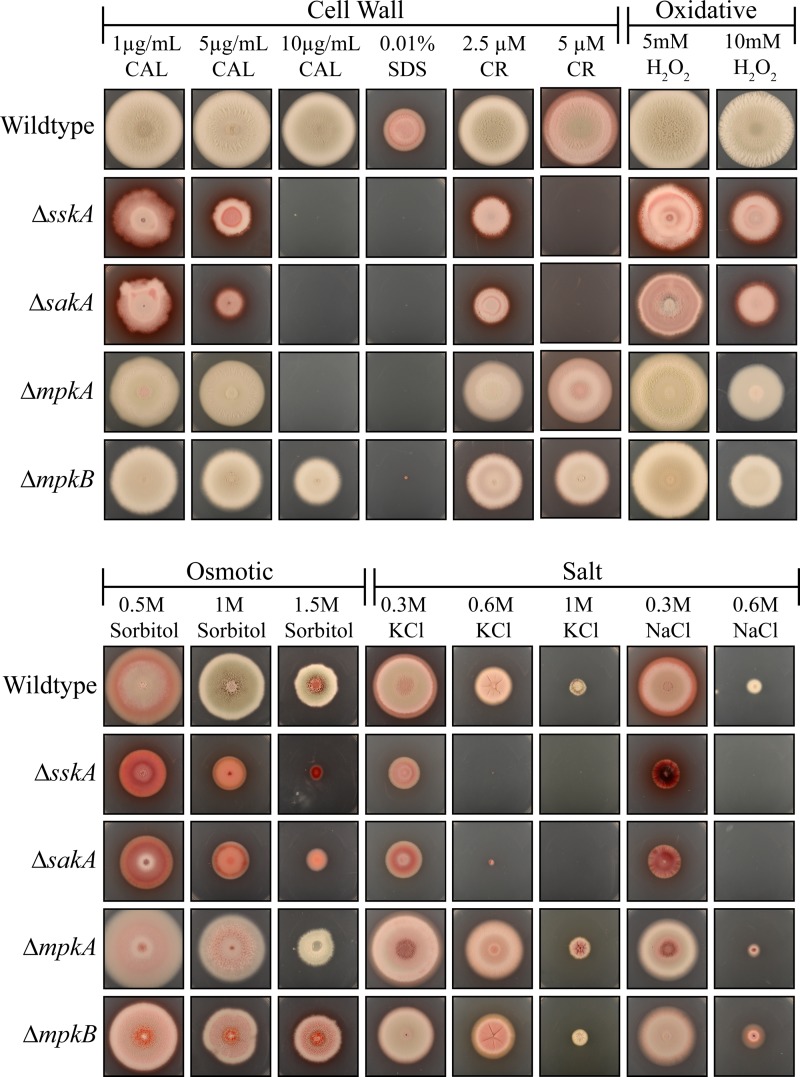
*sskA* and *sakA* are essential for adaptation to cell wall, osmotic, and salt stress. The wild-type, Δ*sskA*, Δ*sakA*, Δ*mpkA*, and Δ*mpkB* strains were grown for 14 days at 25°C on 1% ANM plus GABA plus various stress-inducing agents (calcofluor white [CAL], sodium dodecyl sulfate [SDS], Congo red [CR], hydrogen peroxide [H_2_O_2_], sorbitol, potassium chloride [KCl], and sodium chloride [NaCl]).

The abnormal chitin deposits along hyphae of the Δ*sskA* and Δ*sakA* mutants and the frequent cell lysis of Δ*mpkA* mutant suggested defects in cell wall deposition (Fig. 4). To investigate whether the mutants possess cell wall defects, the wild-type, Δ*sskA*, Δ*sskA sskA^+^*, Δ*sakA*, Δ*mpkA*, Δ*mpkA mpkA^+^*, Δ*mpkB*, and Δ*mpkB mpkB^+^* strains were plated on increasing concentrations of calcofluor white, Congo red, and sodium dodecyl sulfate (SDS) (Fig. 5). Calcofluor white and Congo red both interfere with the construction and stress response of the cell wall by binding to chitin and beta(1-3)glucan, respectively, whereas SDS disrupts the plasma membrane. Compared to the wild type, the Δ*sskA* and Δ*sakA* strains showed increased sensitivity to calcofluor white, SDS, and Congo red (Fig. 5). The Δ*mpkA* strain was sensitive to calcofluor white and SDS but not Congo red (Fig. 5). The Δ*mpkB* strain did not show sensitivity to these cell wall stress agents (Fig. 5). The Δ*sskA sskA^+^*, Δ*mpkA mpkA^+^*, and Δ*mpkB mpkB^+^* strains showed no sensitivity to cell wall stress (see Fig. S1C in the supplemental material).

### SskA and SakA are required for adaptation to high osmolarity, salt stress, and oxidative stress.

Deletion of *T. marneffei* slnA (orthologous to Sln1p) and the gene encoding another hybrid histidine kinase, *drkA*, in *T. marneffei* and other filamentous ascomycetes, results in sensitivity to high-osmolarity conditions (14, 15, 18, 20, 21, 23, 27, 28). Deletion of *T. marneffei* drkA results in increased sensitivity to oxidative stress (23). To investigate whether the deletion of *sskA* in *T. marneffei* also results in sensitivity to high-osmolarity conditions, salt stress, and oxidative stress, the wild-type, Δ*sskA*, Δ*sskA sskA^+^*, Δ*sakA*, Δ*mpkA*, Δ*mpkA mpkA^+^*, Δ*mpkB*, and Δ*mpkB mpkB^+^* strains were plated on media containing different concentrations of sorbitol, potassium chloride (KCl), sodium chloride (NaCl), and H_2_O_2_. In contrast to the wild type and the Δ*mpkA* and Δ*mpkB* mutants and complemented strains, the Δ*sskA* and Δ*sakA* mutants exhibited sensitivity to high concentrations of sorbitol, KCl, and NaCl (Fig. 5; see Fig. S1C in the supplemental material). Compared to the wild type and the complemented strains, growth of the Δ*sskA*, Δ*sakA*, Δ*mpkA*, and Δ*mpkB* strains was slightly reduced on 10 mM H_2_O_2_ (Fig. 5; Fig. S1C).

### SskA acts upstream of SakA during osmotic adaptation.

**FIG 6:**
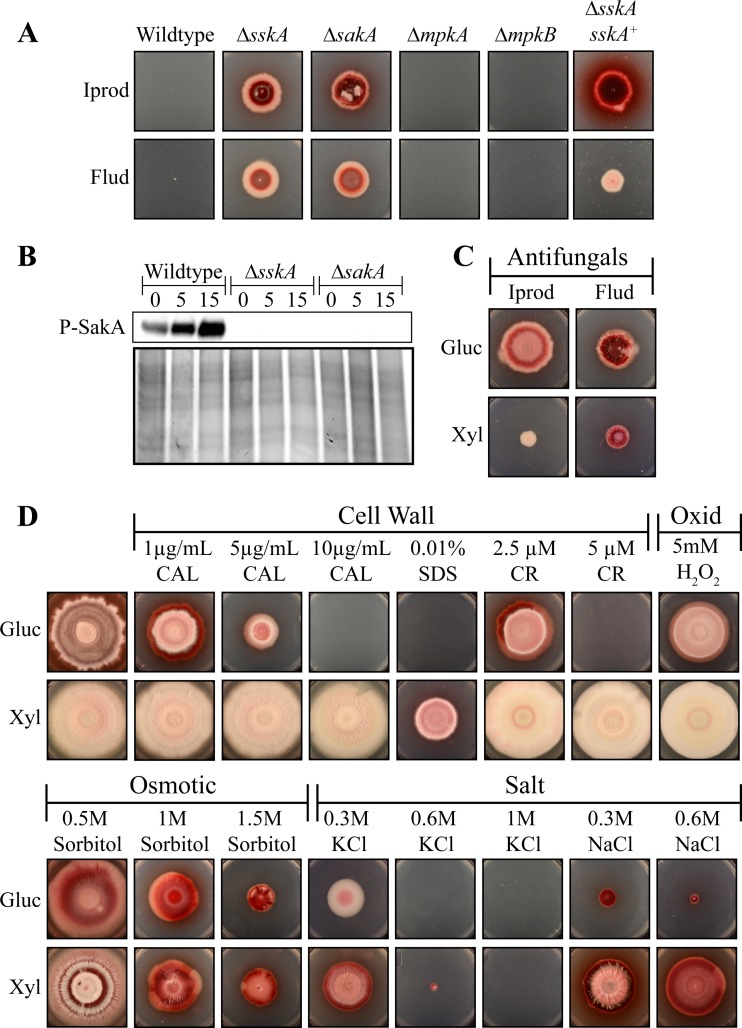
SskA acts upstream of SakA during cell wall, oxidative, osmotic, and salt stress adaptation. (A) The wild-type, Δ*sskA*, Δ*sakA*, Δ*mpkA*, Δ*mpkB*, and Δ*sskA sskA*^+^ strains were grown for 14 days at 25°C on phenylpyrrole (iprodione) or dicarboximide (fludioxonil) fungicides. The Δ*sskA*, Δ*sakA*, and Δ*sskA sskA*^+^ strains show resistance to both iprodione (Iprod) and fludioxonil (Flud). (B) Western blot of phosphorylated SakA (P-SakA) in total denatured protein extracts from the wild-type, Δ*sskA*, and Δ*sakA* strains grown for 2 days at 25°C and transferred to media containing 0.3 M NaCl for 0, 5, or 15 min. (C) Growth after 14 days at 25°C of the Δ*sskA xylP*(*p*)::*sakA^F316L^* strain on dicarboximide (fludioxonil) or phenylpyrrole (iprodione) fungicide and either glucose (noninduced) or xylose (induced). On glucose, the Δ*sskA xylP*(*p*)::*sakA^F316L^* strain exhibits the Δ*sskA* phenotype of resistance to both fludioxonil and iprodione. Growth on xylose, and consequent expression of *xylP*(*p*)::*sakA^F316L^*, partially suppresses this phenotype. (D) Growth after 14 days at 25°C of the Δ*sskA xylP*(*p*)::*sakA^F316L^* strain on various stress-inducing agents (calcofluor white [CAL], sodium dodecyl sulfate [SDS], Congo red [CR], hydrogen peroxide [H_2_O_2_], sorbitol, potassium chloride [KCl], and sodium chloride [NaCl]) and either glucose (noninduced) or xylose (induced).

As the phenotypes of the Δ*sskA* and Δ*sakA* mutants were indistinguishable, we hypothesized that SskA acts upstream of SakA in *T. marneffei*. To test whether SskA may be inducing signaling through the SakA pathway, resistance of strains to the dicarboximide and phenylpyrrole classes of antifungals, fludioxonil and iprodione, was tested. The fungicidal activity of these agents is achieved by induction of the HOG MAPK pathway, as glycerol accumulation in the absence of high external osmolarity results in cell death (17). Deletions of *HOG1* orthologues (SakA) and their upstream activators confer resistance to these antifungals (1, 15, 18, 20, 22, 28–32). As expected, the *T. marneffei* Δ*sakA* mutant is resistant to fludioxonil and iprodione (Fig. 6A). The Δ*sskA* mutant also shows resistance to fludioxonil and iprodione, suggesting that like *drkA*, *sskA* is necessary for induction of the HOG pathway in *T. marneffei* and is acting upstream of SakA (Fig. 6A) (23). The Δ*mpkA* and Δ*mpkB* strains do not show resistance to fludioxonil and iprodione (Fig. 6A). Interestingly, despite showing a wild-type phenotype for all other assays, the Δ*sskA sskA^+^* strain still exhibited resistance to fludioxonil and iprodione (Fig. 6A). This may suggest that wild-type expression is not achieved when *sskA* is integrated at *niaD* and that a reduction in *sskA* expression may be sufficient to result in resistance to these antifungals.

To confirm that SskA acts upstream of SakA in *T. marneffei*, the phosphorylation of SakA was assessed in the Δ*sskA* mutant. Western blot analysis to detect phosphorylated SakA was performed using anti-phospho-p38 antibodies which have been shown to detect phosphorylated SakA homologues in *T. marneffei* and other fungal species (19, 23, 33–37). In the wild type, phosphorylated SakA was detected at 0 min, and the level of phosphorylated protein increased over time (Fig. 6B). No phosphorylated SakA could be detected in either the Δ*sskA* or Δ*sakA* mutant (Fig. 6B).

In addition, the ability of a constitutively active *sakA* allele (*sakA^F316L^*) to suppress the Δ*sskA* mutant phenotypes was assessed*.* The equivalent allele in *S. cerevisiae* Hog1p (F318L) is active, independent of external stimulation by salt and regulation by phosphorylation and the upstream MAPKK Pbs2p (38). As we were unable to successfully transform the Δ*sskA* mutant, the Δ*sskA xylP*(*p*)::*sakA^F316L^* strain (strain G1021) was generated by first transforming strain G816 (Δ*ligD niaD1 pyrG1*) with the *xylP*(*p*)::*sakA^F316L^* construct targeted to *niaD* (strain G1020) and then transforming this strain with the Δ*sskA* deletion construct. Growth of the Δ*sskA xylP*(*p*)::*sakA^F316L^* strain on various stress-inducing agents and either glucose (noninduced) or xylose (induced) was assessed after 14 days of growth at 25°C. On glucose, the Δ*sskA xylP*(*p*)::*sakA^F316L^* strain exhibits a Δ*sskA* phenotype (Fig. 6C and D). Expression of *xylP*(*p*)::*sakA^F316L^* in the Δ*sskA* mutant (growth on xylose) suppressed the decreased conidiation and irregular shaped colonial phenotype (Fig. 6D). In addition, *xylP*(*p*)::*sakA^F316L^* expression in the Δ*sskA* mutant suppressed the sensitivity to cell wall stress, oxidative stress, and NaCl salt stress (Fig. 6D). Growth on xylose did not suppress the Δ*sskA* sensitivity to osmotic or salt stress caused by KCl and only partially suppressed the resistance to fludioxonil and iprodione (Fig. 6C and D).

### *sskA* is essential for morphogenesis of yeast cells both *in vitro* and *ex vivo* at 37°C.

**FIG 7:**
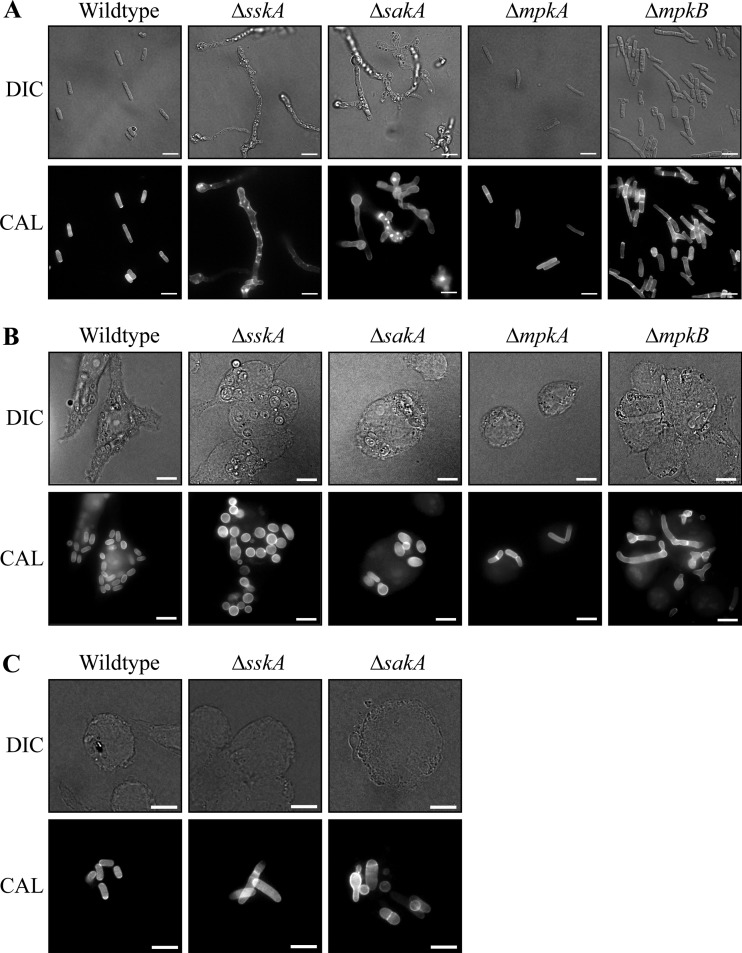
*sskA* and *sakA* are essential for morphogenesis of yeast cells both *in vitro* and *ex vivo* at 37°C. (A) The wild-type, Δ*sskA*, Δ*sakA*, Δ*mpkA*, and Δ*mpkB* strains were grown *in vitro* for 5 days at 37°C on brain heart infusion (BHI) broth. (B) Conidia of the wild-type, Δ*sskA*, Δ*sakA*, Δ*mpkA*, and Δ*mpkB* strains were used to infect J774 murine macrophages, and growth was assessed 24 h postinfection. (C) Conidia of the wild-type, Δ*sskA*, and Δ*sakA* strains were used to infect J774 murine macrophages, and growth was assessed 48 h postinfection. Images were captured using differential interference contrast (DIC) or with epifluorescence to observe fungal cell walls stained with calcofluor white (CAL). Bars, 10 µm.

To examine yeast cell morphogenesis *in vitro*, the wild-type, Δ*sskA*, Δ*sskA sskA^+^*, Δ*sakA*, Δ*mpkA*, Δ*mpkA mpkA^+^*, Δ*mpkB*, and Δ*mpkB mpkB^+^* strains were incubated at 37°C (Fig. 7A). After 5 days at 37°C, wild-type conidia have germinated to produce polarized arthroconidiating hyphae that are fragmenting to liberate uninucleate yeast cells. In contrast, the Δ*sskA* and Δ*sakA* mutants produced aberrant, swollen cells with few septa and numerous abnormal deposits of chitin (Fig. 7A). No yeast cells were observed in the Δ*sskA* and Δ*sakA* strains. The Δ*mpkA*, Δ*mpkB*, and complemented strains were indistinguishable from the wild type (Fig. 7A and data not shown).

To assess the roles of *sskA*, *sakA*, *mpkA*, and *mpkB* during *ex vivo* growth, conidia of the wild-type, Δ*sskA*, Δ*sskA sskA^+^*, Δ*sakA*, Δ*mpkA*, Δ*mpkA mpkA^+^*, Δ*mpkB*, and Δ*mpkB mpkB^+^* strains were used to infect murine J774 macrophages. After 24 h, macrophages infected with wild-type conidia contain numerous yeast cells dividing by fission (Fig. 7B). All strains produced numerous intracellular yeast cells after 24 h. In contrast to the wild type and the Δ*sskA sskA^+^* strain, yeast cells produced by the Δ*sskA* and Δ*sakA* strains showed a rounder and more swollen cellular morphology (Fig. 7B). The average size of Δ*sskA* cells (19.3 ± 0.48 µm^2^) and Δ*sakA* (18.6 ± 0.90 µm^2^) cells significantly increased compared to the wild-type cells (14.6 ± 0.72 µm^2^). This increase in size is statistically significant (Materials and Methods). Yeast cells produced by the Δ*mpkB* strain were misshapen and branched but only when conidia used for infections were isolated from plates containing ammonium, not plates containing GABA (Fig. 7B).

After a longer period of incubation (48 h), unlike the wild type, macrophages infected with the Δ*sskA* and Δ*sakA* strains contained yeast cells with a mixed population of morphologies, including round swollen cells as at 24 h, in addition to long, aberrantly shaped cells (Fig. 7C). A proportion of Δ*sskA* and Δ*sakA* cells also stained very poorly with calcofluor white, and cell lysis was evident (Fig. 7C).

## DISCUSSION

### Role of two-component signaling during asexual development.

Deletion of three of the seven HHKs encoded by *T. marneffei* genes, *drkA* (class III), *slnA* (class VI), and *hhkA* (class X), results in decreased asexual development, suggesting that there are numerous environmental signals influencing this process (23, 39). The *T. marneffei* Δ*sskA* mutant also showed a reduction in asexual development (conidiation), supporting the hypothesis that it is acting downstream of HHKs to regulate this process. The reduction in conidiation in the Δ*sskA* mutant was not as severe as previously noted for the Δ*drkA* mutant (23), suggesting that the other canonical RR in *T. marneffei*, SrrA, is also likely to be involved in regulating asexual development. In *A. nidulans*, both the *SSK1* and *SKN7* homologues have been shown to regulate asexual development (28). Reduced conidiation in the *T. marneffei* Δ*sskA* mutant can be attributed to a lack of SakA phosphorylation, as expression of a constitutively active *sakA* allele (*sakA^F316L^*) in the Δ*sskA* mutant can suppress this phenotype. This suggests that SskA and SakA regulate the activity of one or more transcription factors required to regulate asexual development in *T. marneffei*. This could be a homologue to *S. cerevisiae* Sko1p, Smp1p, or Msn2/4p. Similar to deletion of *sskA*, deletion of the Msn2/4p homologue in *T. marneffei*, *hgrA*, results in an irregular pattern of conidiation in the center of the colony and as a ring around the colony center (41).

In addition, like the Δ*drkA* mutant, Δ*sskA* conidia exhibited reduced viability, especially at 37°C. Deletion of the SskA orthologue in *A. nidulans* also results in decreased conidial viability, and this is due to a lack of SakA phosphorylation (28, 36). The reduction in viability in *T. marneffei* is also likely due to a lack of SakA phosphorylation, as the Δ*sakA* strain showed an identical reduction in conidial viability at both 25°C and 37°C. In *A. nidulans*, phosphorylated SakA accumulates in the nucleus during normal spore development and is proposed to maintain dormancy and/or prevent germination (42). SakA is dephosphorylated during germination, and constitutive phosphorylation of SakA using fludioxinil treatment results in a failure to germinate (42). Phosphorylated SakA interacts with the AtfA nuclear protein in conidia to regulate the expression of genes required for spore viability (42).

### SskA acts upstream of SakA during osmotic adaptation in *T. marneffei.*

The Δ*sskA* mutant displays sensitivity to osmotic and salt stress consistent with the postulated role of SskA, acting downstream of *drkA* and *slnA* in regulating osmotic adaptation. Deletion of the *sskA* orthologues in *A. nidulans*, *Cryptococcus neoformans*, *Cochliobolus Heterostrophus*, and *Candida albicans* also results in sensitivity to osmotic stress (22, 30, 43), albeit only partial sensitivity for *C. albicans* (27, 44). In *C. albicans*, *SSK1* also appears to have a more important role during adaptation to oxidative stress (45). The resistance of the *T. marneffei* Δ*sskA* mutant to the dicarboximide and phenylpyrrole classes of antifungals indicates that SskA is necessary for the phosphorylation of SakA in response to osmotic stress. This was confirmed by showing that no phosphorylated SakA was detected in the Δ*sskA* mutant. In further support, a constitutively active *sakA* allele (*sakA^F316L^*) can suppress the Δ*sskA* NaCl-based salt stress sensitivity and partially suppress the resistance to fludioxonil and iprodione. However, this allele was unable to suppress the Δ*sskA* sensitivity to sorbitol or salt stress caused by KCl. High NaCl concentrations result in ionic imbalance, as Na^+^ competes with K^+^ for uptake, resulting in K^+^ deficiency within the cytosol. In addition, enzymes with K^+^ binding sites can be inhibited by Na^+^. High KCl lowers the cellular osmotic potential and restricts the uptake of water. Therefore, the suppression of the NaCl- but not KCl-based salt stress sensitivity suggests that active SskA is directly affecting ionic homeostasis. This may also indicate that SskA interacts with additional factors to regulate the response to high osmolarity or that a cyclic transition of SakA from the phosphorylated to nonphosphorylated state may be crucial for the response to high osmolarity. It is also possible that some functions of *T. marneffei* SakA are dependent on phosphorylation and interaction with the upstream MAPKK, as the equivalent mutation in *S. cerevisiae* Hog1p (F318L) is active independent of regulation by phosphorylation and Pbs2p (38).

### SskA is necessary for the integrity of the cell wall.

Like the Δ*drkA* mutant, the Δ*sskA* mutant exhibited abnormal chitin deposits along hyphae and showed sensitivity to cell wall stressing agents, indicating defects in cell wall deposition (23). This phenotype is most likely due to a lack of phosphorylated SakA, as the Δ*sakA* mutant showed a phenotype indistinguishable from the phenotype of the Δ*sskA* mutant. In addition, the expression of a constitutively active *sakA* allele could suppress the sensitivity to cell wall stress exhibited by the Δ*sskA* mutant. In *C. albicans*, *ssk1* is required for the expression of a number of genes encoding cell wall biosynthetic enzymes or proteins that regulate cell wall biosynthesis via Hog1 phosphorylation (45). In *S. cerevisiae*, the Msn2/4p transcription factors, which are in part responsible for the induction of genes required for osmotic stress, are activated by phosphorylated Hog1p (11). Many filamentous fungi studied thus far do not have a clear Msn2/4p orthologue, and it remains unclear as to whether the general stress response mediated by Msn2/4 in *S. cerevisiae* is in fact conserved in filamentous fungi (41). The closest homologue in *T. marneffei* is *hgrA* which is not required for adaptation to osmotic stress but has roles specific to hyphal growth, including regulating cell wall biosynthesis and cell wall remodeling (41). It is therefore possible that the cell wall defects in the Δ*sskA* mutant may arise from an inability to activate HgrA via SakA.

It is also possible that SskA is influencing the phosphorylation levels of the MpkA MAPK in response to cell wall stress. However, the sensitivity of the Δ*mpkA* mutant to calcofluor white and SDS is much milder than those of the Δ*sskA* and Δ*sakA* mutants, and no sensitivity to Congo red was observed. In addition, the expression of a constitutively active *sakA* allele could suppress the sensitivity to cell wall stress exhibited by the Δ*sskA* mutant, suggesting that it is the lack of SakA phosphorylation that is the main factor contributing to the cell wall sensitivity of the Δ*sskA* mutant. It is likely that the other canonical RR in *T. marneffei*, SrrA, is also involved in regulating aspects of the cell wall. In *A. nidulans*, deletion of *srrA* results in sensitivity to cell wall stress (28). In addition, putative SrrA binding sites can be identified in the promoters of *T. marneffei* genes required for α-1,3-glucan synthesis (*agsA* and *agsB*), as well as chitin biosynthesis (homologues to *S. cerevisiae* Chs5p and Chs3p).

### Roles of *sskA* during yeast morphogenesis and infection.

Yeast cells produced by the Δ*sskA* and Δ*sakA* strains showed a swollen and aberrant cellular morphology and frequently lysed. This phenotype is predicted to result in an attenuation of virulence in an animal host, although this has yet to be tested. In *C. neoformans*, virulence correlates directly with the level of activity of the HOG MAPK pathway (33). The *hog1* (*sakA* orthologue) gene is crucial for regulating virulence factors such as capsule size and melanin production in virulent serotypes but not in less virulent serotypes (33). Interestingly, the phosphorylation kinetics and localization pattern of Hog1 differ between virulent serotypes and less virulent serotypes and other yeasts (33). This suggests that small alterations in signaling through the conserved two-component and HOG pathway may provide pathogenic fungi with an opportunity to increase their virulent potential. Ssk1 and Hog1 are also essential for virulence and the colonization of the gastrointestinal tract during infection by *C. albicans* (40, 44, 46). In *C. albicans*, the Δ*ssk1* mutant is killed more efficiently by human polymorphonuclear neutrophils (PMNs). This decrease in Δ*ssk1* survival is postulated to be due to increased sensitivity to human defensin-1, a nonoxidative antimicrobial peptide produced by the PMNs, and an increase in the release of PMN cytokines and chemokines during the inflammatory response (47). However, there are likely to be numerous factors contributing to reduced growth during infection. In addition to genes required for osmotic adaptation, Ssk1p and Hog1p in *S. cerevisiae* independently induce the expression of genes required for hypoxic growth (low oxygen), which also occurs in the host environment (48). Hypoxic conditions lead to a gradual increase in Hog1p phosphorylation, and both Ssk1p and Hog1p are required for induced expression of 33 genes contributing to the maintenance of cell integrity during hypoxia (48).

Interestingly, unlike deletion of *drkA*, deletion of *sskA* in *T. marneffei* did not result in hyphal growth during macrophage infection (23). This suggests that the second canonical RR in *T. marneffei*, SrrA, is likely responsible for directly regulating the expression of genes required for the dimorphic switch. As the changes in gene expression required for the dimorphic switch remain unknown, this opens up an exciting avenue for future research.

## MATERIALS AND METHODS

### Molecular techniques and plasmid construction.

*T. marneffei* genomic DNA was isolated as previously described (49). Southern and Northern blotting was performed with Amersham Hybond N+ membrane and [α-^32^P]dATP-labeled probe hybridization by standard methods (50). Total denatured proteins were extracted using a FastPrep-24 sample preparation system (MP Biomedicals) by standard methods (50). SDS-polyacrylamide gel electrophoresis using 50 µg of protein per sample was performed with Tris-glycine-buffered 10% Bio-Rad Mini-PROTEAN TGX stain-free precast gels. Western blotting was performed with Immobilon-P polyvinylidene difluoride (PVDF) filters (Millipore) according to the manufacturer’s instructions. Phosphorylated SakA was detected using phospho-p38 MAPK rabbit antibodies (Cell Signaling Technology). Anti-rabbit IgG horseradish peroxidase (HRP)-linked antibody (Cell Signaling Technology) was used as the secondary antibody and was detected using Clarity Western enhanced chemiluminescence (ECL) substrate (Bio-Rad). Western blot signals were detected by using a Bio-Rad ChemiDoc MP imaging system and Image Lab 5.2.

Sequences of the primers are provided in Table S1 in the supplemental material. A PCR product encompassing *T. marneffei* sskA (PMAA_079660) was generated with primers QQ65 and QQ66 and cloned into pGEM-T Easy to generate pLS7840. To make the pLS7854 deletion construct, a Gateway reaction was performed with pHW7711 (*pyrG^+^*) and an inverse PCR-generated product from pLS7840 using the primers QQ67 and QQ68 (51). The complementation construct pKB7937 was generated by cloning a SacII/SpeI fragment from pLS7840 into pHB7615 (*niaD* targeting pBluescript II SK^+^). The *mpkA* gene was cloned into pGEM-T Easy (pKB7779) using primers PP30 and PP31. Then, the XhoI/EcoRV fragment from pKB7779 was cloned into XhoI/StuI-digested pLitmus29, generating pKB7780. The deletion construct pKB7806 was generated by cloning an XbaI/EcoRI *pyrG* fragment cloned into the XbaI/EcoRI sites of pKB7780. The *mpkA* gene was also amplified using primers NN67 and NN68 and cloned into pGEM-T Easy, generating pLS7830. The complementation construct pLS7831 was generated by cloning a SpeI/NotI fragment from pLS7830 into pLS7804 (51). The *mpkB* gene was cloned into pGEM-T Easy (pKB7719) using primers NN47 and NN48. The deletion construct pKB7721 was generated by cloning a BamHI/EcoRV *pyrG* fragment into BamHI/EcoRV-digested pKB7719. The complementation construct pLS7825 was made by cloning a PCR product generated with primers OO80 and OO81 into pLS7804 (51). The *sakA* gene was amplified using primers PP26 and PP27 and cloned into pGEM-T Easy, generating pLS7808. The *sakA^F316L^* mutation was introduced by performing inverse PCR using primers UU51 and UU52 to generate pKB8002. A PstI/SalI fragment containing the inducible promoter *xylP*(*p*) was cloned into PstI/XhoI pHB7615 (*niaD^t^*) to generate pKB7996. The PstI/SacII *sakA^F316L^* fragment from pKB8002 was cloned into PstI/SacII pKB7996, generating pKB8003 [*xylP*(*p*)::*sakA^F316L^ niaD^t^*]. The deletion construct was generated by overlap PCR using the primers psak-F1 (F stands for forward) and psak-R2 (R stands for reverse) and PCR products generated from the pGEM-T Easy clone and pALX223 with primers psak-F1 and psakR1 (5′ region), psak-F2 and psak-R2 (3′ region), and pyr-F and pyr-R (*pyrG*). The deletion cassette was cloned into pGEM_T Easy, generating pCC7859.

10.1128/mSphere.00086-15.2 Table S1  Oligonucleotides used in this study Download Table S1, DOC file, 0.04 MB. Copyright © 2016 Boyce et al. 2016 Boyce et al. This content is distributed under the terms of the Creative Commons Attribution 4.0 International license.

### Fungal strains and media.

Transformation was performed using the previously described protoplast method (49). Strains used in this study are listed in Table S2 in the supplemental material. The Δ*sskA pyrG^+^* strain (G991), Δ*mpkA pyrG^+^* strain (G1014), Δ*mpkB pyrG^+^* strain (G988), and Δ*sakA pyrG^+^* strain (G990) were generated by transformation of strain G816 (Δ*ligD niaD1 pyrG1*) with linearized deletion constructs pLS7854, pKB7806, pKB7721, and pCC7859 and selecting for PyrG^+^. The Δ*sskA sskA^+^* strain was generated by transforming strain G816 (Δ*ligD niaD1 pyrG1*) with the pKB7937 plasmid and selecting for *niaD*^+^. This strain (G1018) was transformed with the linearized *sskA* deletion construct pLS7854, generating strain G1019. This strain was plated on 10 mM chlorate to select for the loss of the *sskA^+^* plasmid integrated at *niaD.* The *xylP*(*p*)::*sakA^F316L^* Δ*sskA* strain (G1021) was generated by transforming strain G816 (Δ*ligD niaD1 pyrG1*) with the pKB8003 plasmid and selecting for *niaD*^+^. This strain (G1021) was transformed with the linearized *sskA* deletion construct pLS7854, generating strain G1021. The Δ*mpkA pyrG*^−^ and Δ*mpkB pyrG*^−^ strains (Table S2) were generated by plating on medium containing 1 mg/ml 5-fluoroorotic acid (5-FOA) supplemented with 10 mM (NH_4_)_2_SO_4_ and 5 mM uracil to select for the loss of the *pyrG* marker. These strains are unable to grow in the absence of 5 mM uracil. The Δ*mpkA* and Δ*mpkB* deletion mutants were complemented by transformation of the *pyrG^−^* strains with pLS7831 (*mpkA*) and pLS7825 (*mpkB*) and selecting for *pyrG^+^*. Gene deletion and the integration of the complementation constructs at *pyrG* or *niaD* was confirmed by Southern blot analysis of genomic DNA.

10.1128/mSphere.00086-15.3 Table S2  Strains used in this study Download Table S2, DOC file, 0.05 MB. Copyright © 2016 Boyce et al. 2016 Boyce et al. This content is distributed under the terms of the Creative Commons Attribution 4.0 International license.

To test the stress resistance of mutants, strains were grown at 25°C and 37°C on *A. nidulans* minimal medium (ANM) with 1% glucose and 10 mM GABA and supplemented as follows: for salt stress using 0.3, 0.6, and 1 M KCl and 0.3 and 0.6 M NaCl; for osmotic stress using 0.5, 1, and 1.5 M sorbitol; for oxidative stress using 5 and 10 mM H_2_O_2_; for cell wall stress using 0.1, 5, and 10 µg/ml calcofluor white, 2.5 and 5 µM Congo red, and 0.01% SDS; for resistance using 30 µg/µl iprodione and 10 µg/µl fludioxonil. All samples were incubated for 14 days.

### Macrophage assay.

J774 murine macrophages (1 × 10^5^) were seeded into each well of a six-well microtiter tray containing one sterile coverslip and 2 ml of complete Dulbecco’s modified Eagle medium (complete DMEM) (DMEM, 10% fetal bovine serum, 8 mM l-glutamine, and penicillin-streptomycin). Macrophages were incubated at 37°C for 24 h before activation with 0.1 µg/ml lipopolysaccharide (LPS) from *E. coli* (Sigma) and incubated a further 24 h at 37°C, washed in phosphate-buffered saline (PBS) and 2 ml of complete DMEM or RPMI 1640 medium containing 1 × 10^6^ conidia was added. A control lacking conidia was also performed. Macrophages were incubated for 2 h at 37°C (to allow conidia to be engulfed), washed once in PBS (to remove free conidia), and incubated a further 24 or 48 h at 37°C. Macrophages were fixed in 4% paraformaldehyde and stained with 1 mg/ml fluorescent brightener 28 (calcofluor white [CAL]) to observe fungal cell walls. Mounted coverslips were examined using differential interference contrast (DIC) and epifluorescence optics for cell wall staining and viewed on a Reichart Jung Polyvar II microscope. Images were captured using a SPOT charge-coupled-device (CCD) camera (Diagnostic Instruments Inc.) and processed in Adobe Photoshop. The numbers of ungerminated conidia, germlings, or yeast cells were recorded in a population of approximately 100 in three independent experiments. Mean and standard error of the mean values were calculated using GraphPad Prism3. Unpaired *t* tests were performed on the mean and standard error of the mean values to test statistical significance (95% confidence interval).
