# Supplementary figures and images for: Two-Component Signaling Regulates Osmotic Stress Adaptation via SskA and the High-Osmolarity Glycerol MAPK Pathway in the Human Pathogen Talaromyces marneffei
Source: mSphere. 2016 Feb 24;1(1):e00086-15. doi: 10.1128/mSphere.00086-15 (PMC4863612; doi:10.1128/mSphere.00086-15)

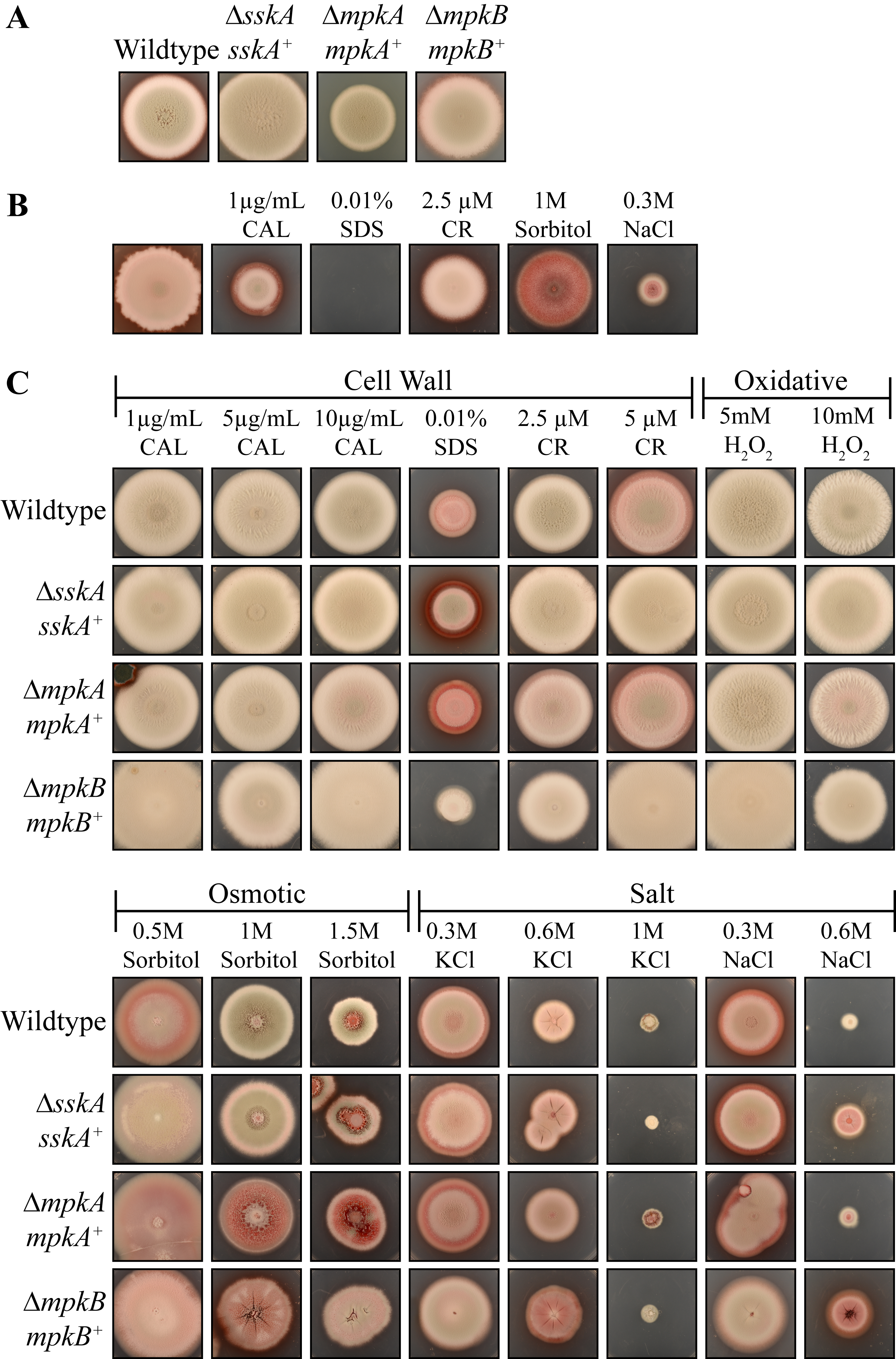

Supplement: Figure S1 [file sph001162026sf1.tif]
