# Supplementary material for: Two-Component Signaling Regulates Osmotic Stress Adaptation via SskA and the High-Osmolarity Glycerol MAPK Pathway in the Human Pathogen Talaromyces marneffei
Source: mSphere. 2016 Feb 24;1(1):e00086-15. doi: 10.1128/mSphere.00086-15 (PMC4863612; doi:10.1128/mSphere.00086-15)
Supplement: Table S1 [file sph001162026st2.doc]

**Table S1. Oligonucleotides used in this study**

| Gene | Number | Sequence |
| --- | --- | --- |
| *sskA* | QQ65 | AAGCCAGTGGACCAAGGTG |
|  | QQ66 | ACAACGACATCAAAAGCGG |
|  | QQ67 | GGGGACCCAGCTTTCTTGTACAAAGTGGTCATTGATAAACGGTGTGTG |
|  | QQ68 | GGGGAGCCTGCTTTTTTGTACAAACTTGTCTCGATGGTGCCGGATGA |
| *mpkA* | PP30 | GCCCGTATCTTTTCTATCCC |
|  | PP31 | AGGGCGCTGGCGACTTTTCC |
|  | NN67 | ATGAGCTCTCACTCCAGTTAAGCACG |
|  | NN68 | GCACCAGGAGAATGACGG |
| *mpkB* | NN47 | GAACGATTGAACCTGGAACC |
|  | NN48 | CTTCTGCGTATGCGGGACTG |
|  | OO80 | AAGAGCTCCGACTGGCGTGTGTAATG |
|  | OO81 | CCAGATCTTGTGATTGCCAGGTTAGC |
| *sakA* | PP26 | GAGGGATGATGTAGAGGCAG |
|  | PP27 | CCTAAACGTGATCTCAGCAG |
|  | UU51 | TGAGAAGTTAGACTGGTCGT |
|  | UU52 | TCGGCAACTGGTTCGTCG |
|  | psak-F1 | GTGACATACCATTCTCGGGGGCTCG |
|  | psak-R1 | TGTGTAACGGCAACCGGGCTGCTC |
|  | psak-F2 | GCATTGTCTGCGGTGAAGATCATGAC |
|  | psak-R2 | GAGAGGTTCTATACCAAGGTACGTG |
|  | pyr-F | TTGCCGTTACACATTTCCACTCAC |
|  | pyr-R | CGCAGACAATGCTCTCTATCC |
