# Supplementary material for: Two-Component Signaling Regulates Osmotic Stress Adaptation via SskA and the High-Osmolarity Glycerol MAPK Pathway in the Human Pathogen Talaromyces marneffei
Source: mSphere. 2016 Feb 24;1(1):e00086-15. doi: 10.1128/mSphere.00086-15 (PMC4863612; doi:10.1128/mSphere.00086-15)
Supplement: Table S2 [file sph001162026st3.doc]

**Table S2. Strains used in this study**

| Number | Strain name | Genotype | Source |
| --- | --- | --- | --- |
| G816 | ∆*ligD pyrG^-^* | ∆*ligD niaD pyrG* | 1 |
| G944 | ∆*ligD pyrG^t^* | ∆*ligD niaD pyrG* [*pyrG^t^ SK^+^*] | 2 |
| G991 | Δ*sskA pyrG^+^* | ∆*ligD niaD pyrG* Δ*sskA::pyrG^+^* | This study |
| G1018 | *sskA^+^* | ∆*ligD pyrG* [*niaD^t^ sskA^+^*] | This study |
| G1019 | Δ*sskA sskA^+^* | ∆*ligD pyrG* Δ*sskA::pyrG^+^* [*niaD^t^ sskA^+^*] | This study |
| G1020 | *xylP(p):: sakA^F316L^* | ∆*ligD pyrG* [*niaD^t^ xylP(p)::sakA^F316L^*] | This study |
| G1021 | Δ*sskA xylP(p):: sakA^F316L^* | ∆*ligD pyrG* Δ*sskA::pyrG^+^* [*niaD^t^ xylP(p)::sakA^F316L^*] | This study |
| G990 | Δ*sakA* *pyrG^+^* | ∆*ligD niaD pyrG* Δ*sakA::pyrG^+^* | This study |
| G1014 | Δ*mpkA* *pyrG^+^* | ∆*ligD niaD pyrG* Δ*mpkA::pyrG^+^* | This study |
| G958 | Δ*mpkA* *pyrG^-^* | ∆*ligD niaD pyrG* Δ*mpkA* | This study |
| G959 | Δ*mpkA* *mpkA^+^* | ∆*ligD niaD pyrG* Δ*mpkA* [*pyrG^t^ mpkA^+^*] | This study |
| G988 | Δ*mpkB* *pyrG^+^* | ∆*ligD niaD pyrG* Δ*mpkB::pyrG^+^* | This study |
| G961 | Δ*mpkB* *pyrG^-^* | ∆*ligD niaD pyrG* Δ*mpkB* | This study |
| G1022 | Δ*mpkB mpkB^+^* | ∆*ligD niaD pyrG* Δ*mpkB* [*pyrG^t^ mpkB^+^*] | This study |

1. **Bugeja HE, Boyce KJ, Weerasinghe H, Beard S, Jeziorowski A, Pasricha S, Payne M, Schreider L, Andrianopoulos A.** 2012. Tools for high efficiency genetic manipulation of the human pathogen *Penicillium marneffei*. Fungal Genet Biol **49:**772–778. http://dx.doi.org/10.1016/j.fgb.2012.08.003.

2. **Boyce KJ, McLauchlan A, Schreider L, Andrianopoulos A.** 2015. Intracellular

growth is dependent on tyrosine catabolism in the dimorphic fungal pathogen *Penicillium marneffei*. PLoS Pathog **11:**e1004790. http://dx.doi.org/10.1371/journal.ppat.1004790.
